# Investigating Employment Quality for Population Health and Health Equity: A Perspective of Power

**DOI:** 10.3390/ijerph19169991

**Published:** 2022-08-13

**Authors:** Kaori Fujishiro, Emily Q. Ahonen, Megan Winkler

**Affiliations:** 1 Division of Field Studies and Engineering, National Institute for Occupational Safety and Health, Centers for Disease Control and Prevention, Cincinnati, OH 45226, USA; 2 Division of Occupational and Environmental Health, Department of Family and Preventive Medicine, School of Medicine, University of Utah, Salt Lake City, UT 84132, USA; 3 Department of Behavioral, Social and Health Education Sciences, Rollins School of Public Health, Emory University, Atlanta, GA 30322, USA

**Keywords:** occupational safety and health, health disparities, social determinant of health, employment conditions, employment quality, precarious employment, institution, social policy, power resource

## Abstract

Employment quality (EQ) has gained increasing attention as a determinant of health, but the debate among occupational health researchers over the measurement of EQ poses a challenge to advancing the literature. This is especially problematic when the concept is used across social, cultural, and national borders, as EQ is shaped by power dynamics within sociopolitical and economic contexts that are specific to each society. Investigating EQ in context could help develop a clearer understanding as to why EQ is configured in certain ways, how best EQ could be measured, how EQ impacts health, and ultimately how EQ could be improved. In this paper, we propose that attention to social context—and in particular power—may help advance the research on EQ and health. We present an allegory, or a visual description, that articulates the power balance in the employer–worker relation as well as in the sociopolitical context in which the employer–worker relation takes place. We end by proposing specific approaches for occupational health researchers to incorporate a perspective of power in EQ research that may clarify the concept and measurement of EQ. A clearer recognition of EQ as a product of power in social context aligns with the research approach of addressing work as a social structural determinant of health.

## 1. Introduction

The health impacts of work have long been recognized. Occupational medicine and industrial hygiene, the main disciplines concerned with work-related health, have historically acknowledged that economic and structural aspects of society—including the relation between labor and capital—create health and ill-health of the working population [1,2]. However, the research and practice of occupational safety and health in recent decades have mostly focused on hazard exposure and its direct link to injury or physiologic link to illness. This approach sees work as a setting in which hazard exposure—be it physical, chemical, biological, or psychosocial—occurs and people experience subsequent ill-health, but it does not foster an understanding of work as a social institution embedded in a broader social structure [3]. Because the discipline of occupational health and safety has not amply equipped itself for addressing social contexts outside of the workplace, occupational health researchers face a conceptual and methodological challenge as they try to investigate the changing nature of work as it relates to health. In this paper, we underscore work as a social structural determinant of health and explore ways for occupational safety and health to incorporate *power*—an important feature of every society.

We specifically focus on employment quality (EQ), an aspect of work that has gained increasing attention as a determinant of health [4,5,6]. *Employment* refers to “the legal relationship that […] determines the obligations, responsibilities, and expectations of employers and employees” [7] (p. 306); thus, EQ captures the nature of these contractual and relational features of work [4,5,6,8,9], represented by dimensions such as *employment stability* (e.g., the type and length of contract), *material rewards* (e.g., pay and benefits), *working time arrangements* (e.g., the length and predictability of work hours), *training and employability opportunities* (e.g., on-the-job training), *worker*’ *rights and protections* (e.g., access to unemployment insurance), *empowerment and access to collective organizations* (e.g., union representation), and *interpersonal power relations* (e.g., intimidation, discrimination) [10]. EQ is distinct from the characteristics of work tasks, such as the level of mental concentration required, the pace at which tasks are performed, and the need for handling hazardous materials. Research on these aspects, sometimes referred to as “work-task intrinsic characteristics” [6] or “work quality” [11,12], have a longer history than EQ because occupational health researchers in the last several decades focused on what workers are exposed to as they perform job tasks [1,2]. Because the way people are attached to their jobs has become diverse [13], research interest has been increasing in how EQ affects the health and well-being of workers [4,5,6,8,9]. In recent years, studies have shown associations between poor EQ (i.e., combinations of undesirable employment conditions measured in various ways) and poor health [4,10,14,15], as well as potential pathways (e.g., material deprivation, stress, occupational hazard exposure [10,16]). However, the lack of consensus as to how to measure EQ dimensions has been recognized as a major challenge in the literature on EQ and health [4,5,6,8].

The interest in EQ is relatively new but rapidly growing in the United States, whereas EQ research has a longer history elsewhere [4,10,14,15]. Adapting the concept of EQ across societies is difficult because work and the health of working people are embedded in historical, sociopolitical, economic, and cultural features of society [17]. These social features determine who does what kind of work, which jobs are more highly rewarded than others, what industries have strong ties to policy making, which workers are contemplated for protection under the law, what social safety net is available to whom and who pays for it [18,19]. EQ research is challenging because EQ is shaped by these complex and intertwined features of society. However, for the same reason, investigating EQ in social contexts could clarify why EQ is shaped in certain ways and how EQ is associated with health.

Recognizing the challenges in the literature, this paper proposes a framework that brings the occupational health researchers’ attention to the social contexts that shape EQ. Drawing from Walter Korpi’s power resource model [20], we pay special attention to power, defined as the capacity of individuals, groups, and institutions to reward and punish others [20]. Researchers interested in EQ and health conceptually acknowledge that “the nature of prevailing employment conditions is affected by the power relationships between employers and employees” [4] (p. 230), yet the current debate over operationalization, or the measurement of EQ as it relates to health does not fully engage in the discussion of power and thus risks neglecting its importance. This paper first briefly summarizes the current research findings on EQ and health, highlighting recent studies from the United States. Then, we describe how incorporating a perspective of power can clarify our understanding of EQ and health using a visual depiction of power both in the social context and in the employer–worker relation. We conclude by proposing next steps for advancing the research on EQ, health, and health equity by incorporating a perspective of power.

## 2. Employment Quality, Health, and Health Equity

Occupational health researchers have investigated EQ mostly in its poor form, *precarious employment*, a cluster of undesirable employment features such as insufficient pay and benefits, long or insufficient work hours, unpredictable work schedules, and high job insecurity. Several reviews [4,10,14,15] reported that precarious employment (i.e., poor EQ) was associated with a host of poor physical health (e.g., worse self-rated general health, increased cardiovascular risk factors, increased cardiovascular deaths) and mental health (e.g., minor psychiatric morbidity, anxiety, depression, suicide ideation) as well as occupational injuries and accidents. More recently, some researchers constructed distinct types of EQ, ranging from very good EQ—represented by secure, high-paying, and autonomous jobs—to very poor EQ, which is similar to precarious employment. Although different measures were used to identify EQ types, these EQ typology studies found that better EQ is associated with better health, and poor EQ is associated with poor health [16,21,22].

In recent years, US researchers started exploring the association between EQ and health in the US context. For example, Patil and colleagues [23] constructed a precarious employment score during pregnancy and found an association between high precarity (i.e., poor EQ) and low birth weight. Peckham and colleagues [16] identified eight EQ types, six among the employed and two among the self-employed. Consistent with studies in Europe and elsewhere, Peckham and colleagues found that, compared with those in poorer EQ types, workers in better EQ types were more likely to report better self-rated general health, better mental health, and lower rates of occupational injuries. In addition, they found suggestive evidence for material deprivation, employment-related stress, and occupational risk factors (e.g., physical hazard exposure) to be potential mediating mechanisms. Some US studies used longitudinal data to identify patterns of EQ experience over the course of the individuals’ working years, and reported that poor and deteriorating EQ over time was associated with poor health [24,25]. Moreover, US studies consistently found that women, people of color, and those with limited education were more likely to experience poor EQ both at a single point in time [23,26] and over their life-course [24,25,27].

The growing literature on EQ and health corresponds with the recognition that EQ is a social determinant of health. The World Health Organization included employment and working conditions as health determinants in its 2011 publication on health equity [8]. Healthy People 2030, the US national objectives for improving health and well-being, recognizes stable employment as a key element of economic stability, one of five social determinants of health [28]. The literature summary for the Healthy People 2030 Social Determinants of Health identifies health implications of employment and underemployment (i.e., involuntary part time, poverty-wage employment, insecure employment, intermittent employment) as future research needs. Conducting surveillance and intervention studies on non-standard employment (i.e., part-time, temporary agency, contract) [29] and precarious work are included in the current strategic plan of the US National Institute for Occupational Safety and Health (NIOSH) [30]. Given such organizational attention, it is important to develop a clear conceptualization and methods of measuring EQ as a determinant of health.

## 3. Power as a Key to Conceptualizing EQ

Although a literature on EQ and health has been growing over 20 years, it has not achieved a consensus on how to conceptualize or measure EQ [5,6]. There is a general agreement on the construct’s multidimensional nature [5]; however, debates continue as to which dimensions are to be included and what indicators best represent each dimension. The difficulty is clearly seen in recent studies from the United States, where the attention to EQ has just started and therefore existing public health surveillance systems and other social surveys are not designed to capture EQ-relevant information. These pioneering US studies explored ways to align available data with EQ dimensions identified in European studies, i.e., [10]. For example, the dimension of *interpersonal power relations* has been operationalized with US data as union membership [25], intimidation about reporting occupational injury and safety concerns [31], the ability to control one’s schedule [16,32], decision-making power and autonomy on the job [16,25,27], experiences of sexual or other types of harassments [16], and forced retirement among older workers [32]. Many of these indicators could represent other EQ dimensions as well, such as *employment stability* (i.e., forced retirement), *work time arrangements* (i.e., schedule control), *collective organization* (i.e., union membership), and *workers*’ *rights* (i.e., reporting safety concerns). Moreover, these dimensions influence each other: employment stability may be related to schedule predictability, an aspect of work time arrangements, if being available on short notice is required for continued employment; and schedule predictability can be related to income volatility (i.e., *material rewards*) if workers are paid hourly [33]. Recognizing the interrelatedness among EQ dimensions, researchers have used statistical techniques, such as latent class cluster analysis [16], multichannel sequence analysis [25], and principal component analysis followed by summary scores using factor loadings as weights [32] to accommodate complexities of EQ dimensions. These efforts, however, do not address *why* the complexities exist.

We hypothesize that EQ dimensions are interconnected because they are underpinned by the power relationship between those who provide labor (i.e., workers) and those who purchase it (i.e., employer). Theoretically, the more power the employer has relative to workers, the poorer EQ could be in any dimension to the extent allowed in the society (e.g., minimum wage, labor regulations, public opinions). The power relationship that shapes EQ is therefore inseparable from the political, legal, economic, and normative powers that are specifically configured within each society [17]. In addition, racism, sexism, and other systems provide the basis for distributing opportunities and resources unevenly through social institutions and interpersonal interactions [34,35], including work and the employer–worker relationship. For example, in a society that values the heteronormative, nuclear form of family and patriarchal gender division of labor, jobs held predominantly by men and by women would have different EQ, men’s and women’s paid work participation would be supported differently [36]. As a result, the association between EQ and health might be patterned by the normative gender role expectations and social policies relevant to families with young children [37]. These various forms of power are often difficult to recognize because they operate across many social institutions to form social customs and habits that are taken for granted and normalized [38].

The ubiquitous nature of power dynamics poses a challenge to conceptualizing and measuring EQ. At this early stage of EQ research in the United States, researchers have prioritized causal inferences, or showing the association between poor EQ and poor health, e.g., [16,31], and identifying who is at risk, e.g., [27,32]. This type of research has introduced and legitimized the concept of EQ in the discussion of occupational and population health in the United States. Research that explores determinants of EQ in specific sociopolitical contexts would further advance the field as most EQ indicators have long been associated with health [4,14,15]. This is an important next step, because by understanding *why* poor EQ exists in the first place, research could shed light on ways to improve EQ, which may lead to better worker health and reduce health inequalities.

## 4. Recognizing Power and Its Social Configuration as It Relates to Health: An Allegory

**Figure 1:**
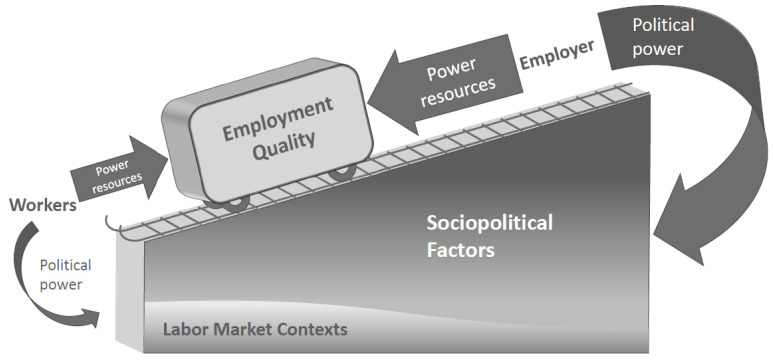
An allegory: Employment quality as a power balance between workers and the employer on an uneven playing field. Informed by Korpi’s Power Resource Model [20].

As many scholars in the field of occupational health have pointed out, employment relations are shaped by power relations [4,8,10,39,40,41,42]; therefore, research on EQ and health would benefit from considering power more explicitly as a determinant of EQ. As an effort to help occupational health researchers incorporate a perspective of power in their research, we developed a visual depiction, or an allegory, of the employer–worker power balance on an uneven playing field (Figure 1). We propose this allegory as a thinking device that makes power visible and challengeable both in the employer–worker relation and in the sociopolitical context in which the employer–worker relation takes place. The depiction is primarily based on the US context because the authors are most familiar with it, but this allegory may be useful in thinking about the ways in which power operates and impacts EQ in any society. In the following sections, we discuss concepts used in the allegory in detail.

### 4.1. Power That Creates an Uneven Playing Field

We first focus on the slope that represents a social context for EQ. In the context of globalization and technological advances, many scholars in economics, sociology, and political science have identified specific drivers of EQ deterioration, particularly in the United States, over the last four decades: the shareholder model that prioritizes gains for investors; public policies that weaken unions; and deregulations that allowed market concentration both as monopoly and monopsony [13,42,43,44,45]. At the same time, cultural shifts toward favoring privatization of public goods (e.g., education, medical care), deregulation, individual responsibility, and flexibility as a “state of mind” [42] (p. 148) have permeated throughout society and legitimized these structural changes. Sectors previously insulated from these mindsets—including nonprofit organizations, academia, and the public sector—have increasingly incorporated these cultural shifts [46,47]. Because these shifts have normalized the structural changes, the structural disadvantage of workers is incrementally made invisible. As a result, it has become difficult to recognize the degree to which employment relations take place on an uneven playing field.

Although these structural and cultural shifts are large in scale, they are not naturally occurring phenomena; therefore, it is important to locate agency and sources of power in these changes. To make power in broader society more visible, we show the slope in Figure 1 as favoring employers. They can directly influence society through mechanisms such as lobbying, political contributions, close ties between public offices and private industries (i.e., “the revolving door”), legal actions, and media discourses [42,48]. In some cases, the sheer size of a corporation is a source of power because of the immense influence its actions can have on the market and government decision-making [49]. In addition to the direct influence on society, common practices among employers can collectively tilt the playing field by changing labor market contexts. Flexible staffing practices (i.e., the use of temporary agencies and contract workers), for example, increase the proportion of the workforce insecurely attached to employment [50], which normalizes such forms of employment and signals to workers that their options are limited. When the employer and the workers both know that secure employment is diminishing, the employer can continue to only offer insecure employment [42].

Workers, too, have sources of power for potentially tilting the playing field in their favor, such as voting, community activism, and social media mobilization. Historically, as Herbert Abrams [1] summarizes, “the worker played a primary role as the basis of every significant improvement in legislation, factory inspection, compensation, correction, and prevention” (p. 56). However, Abrams also points out that the success of the workers’ effort to improve working conditions “has fluctuated with the strength of unionization and the political character of the government in power” (p. 69). Although the second year of a pandemic has occasioned some increase in workers’ power in successful unionization and other collective actions [51], exercising workers’ power is generally difficult because they often lack a structural support such as labor unions or community organizations. Also, because individual responsibility and flexibility—what Heather Scott sees a euphemism for insecurity [42] (p. 145)—have been extensively promoted as social values [42], workers themselves may not see the potential of their power. Consequently, the explicit and implicit power negotiations that determines EQ occur in a social context that strongly favors the employer [8,40].

### 4.2. Power That Operates on an Uneven Playing Field

In a context that favors the employer, workers and employers engage in direct power dynamics that determine EQ. We see EQ as a package of what the employer and workers accept as compensations and conditions for the labor workers provide. Workers’ sources of power to achieve better EQ are education, skills, and unions or other forms of collective organizations. The employer, on the other hand, strives for growth, organizational efficiency, and lower labor costs through tools such as job simplification and flexible staffing, which lead to lower compensation and less stable employment for the workers [50]. The workers’ human capital and collective organization on one hand, and the employer’s power of hiring and firing, job design, relocation and anti-union positions on the other, are countervailing sources of power that place the EQ package at a certain location on a conceptual continuum of better and poorer EQ.

As described in the figure, we propose that the location of EQ is on a playing field that favors employers. To understand power balance on an uneven playing field, Korpi’s power resource model [20] offers a useful framework. Korpi argues that two parties can engage in three possible situations—exchange, conflict, or exploitation—depending on the power they can bring to bear. If the two parties both possess what the other wants and decide to offer it to each other (e.g., workers provide needed labor, and the employer offers desired wages), an exchange occurs. If both parties choose to withhold what the other wants, a conflict occurs (e.g., a labor strike over inadequate wages). Exchange and conflict are likely if the playing field is relatively even; that is, the employer and workers believe that they have more or less equal power. When the playing field is not even and both parties know which way it is tilted, the third situation—exploitation—is likely to occur [20] (p. 35). Since conflicts are costly to both parties (e.g., negative publicity, lost productivity and revenues for the employer, lost wages and potentially lost jobs for workers), if both know that workers lack power (e.g., desired skills, union and public support, legal resources), workers may provide the needed labor even under poor-quality employment.

Power can thus effectively serve the powerful *without being used* as long as all parties share the understanding of how the slope is tilted in a given society [20,38]. That is, actual action and capacity to act, in situations of exploitation, serve the same purpose for the powerful. This shared understanding makes the existing power balance invisible and difficult to incorporate in EQ research. Hourly-paid workers may accept to work long hours to compensate for inadequate pay, and parents of young children may accept part-time work or night shifts because they lack childcare options. These individual behaviors and decision-making occur in particular social, political, and economic structures of power. Because the playing field is tilted, instead of demanding higher pay from the employer or affordable childcare options from policymakers, these workers make personal arrangements to make poor EQ workable for them while the productivity and flexibility of these workers benefit the employer. In Table 1, we summarized the concepts in Figure 1 that are discussed in this and previous sections.

### 4.3. Other Axes of Power That Shape EQ

In addition to power that is directly relevant to employment, various other axes of power in a given society may help clarify the link between EQ and health inequity. In the United States, for instance, social stratification created by sexism and racism systematically place certain groups of people in the employer position with power and certain others in the position of workers with less power [53,54]. Thus, the imbalance of power between employers and workers aligns with other power imbalances such as between men and women or white people and people of color. These alignments facilitate employment to be a relation of exploitation, rather than an exchange or a conflict [20]. Studies by Campos-Serna and colleague [55] and Rosenberg and colleagues [56] offer an illustration: they reported that immigrants and women of color are overrepresented in hospitality, retail, and care work (e.g., home care aides, hotel room cleaners) and that these jobs have generally poor EQ. Another example is the wage decline in jobs as the proportion of women increases. Using the US Census data from 1950 to 2000, Levanon and colleagues [57] presented that changes in a job’s gender composition were followed by changes in the median wage of the job even after controlling for education and experience on the job. They interpreted this finding as the devaluation of work performed by women. The concentration of marginalized people in certain occupations would likely keep EQ low for those jobs, and the poor EQ of these jobs, in turn, would limit workers’ opportunity to gain power and resources to change the situation. This cyclical process, as McCartney and colleagues [58] argue, would perpetuate existing power imbalances. Various consequences of poor EQ, including poor health, are likely to be distributed unevenly among population subgroups with varying degrees of power.

Another important structure of power that shapes EQ is the welfare state, or the system whereby the government ensures the health and well-being of its people [59]. Services needed for this purpose include education; housing; medical care; child, elder, and disability care and support; and income replacement during periods of unemployment, incapacity, illness and recuperation, pregnancy, caregiving, and post retirement. What services are provided to whom, to what extent, and in what way (i.e., universal entitlement, social insurance, or means-tested) vary by society and thus tilt the playing field differently [3]. For example, in the United States, many such services are either provided optionally by the employer (e.g., medical care insurance), or purchased in a market with the income from employment (e.g., childcare), which can limit the workers’ ability to reject poor EQ jobs, accustoms them not to expect much from the social safety net, and erodes the concept of collective action for the public good [60,61]. All of these contribute to the existence of poor EQ in society.

### 4.4. Understanding EQ and Health from a Perspective of Power

Through the use of this allegory, we have come to conceptually understand EQ as a product of power imbalance between workers and the employer in sociopolitical and economic contexts that favor the employer. Since EQ has been closely associated with health [4,10,14,15], understanding EQ from a perspective of power may help advance the literature on EQ and health. The power dynamics depicted above the slope in Figure 1 may be more or less applicable across contexts, but what forms the slope and sets the grade (i.e., the degree of favoring the employer or workers) would vary considerably in different societies at different times.

Not specific to EQ, many prominent models of occupational health and health inequity have directly incorporated power [39,40,41]. Lipscomb and colleagues [39] pointed out that policies and institutional factors that give employers advantages must be made explicit “or they may be embedded in social norms” (p. 43). Muntaner and colleagues [40] proposed a macro-level model of employment relations and health inequalities with a clear focus on “the reciprocal relation between political power [of labor unions, corporations, political parties, and civic organizations] and policy making” (p. 218) that determines employment conditions. Schnall and colleagues [41] identified that understanding how the macro-economic environment changes the nature of work is one of the important research areas to pursue. Sorensen and colleagues [52] also recently expanded their earlier conceptual model for worker health and safety to include social, political, and economic contexts as well as employment and labor patterns. They encourage researchers to examine these concepts as forces influencing work, safety, health, and well-being. Despite these repeated calls for addressing power in occupational health research, empirical studies that directly investigate power remain scarce [62,63]. In the next section, we describe approaches to incorporate the concept of power in EQ research. They are largely an appeal for greater attention to power in studying EQ as a determinant of health, rather than specific recommendations for testing the relationships and assumptions put forth in our allegory. Given the current limited focus on power in occupational health research, we believe these research approaches provide the most feasible and important immediate next steps.

## 5. EQ research Approaches for Incorporating a Perspective of Power

To move EQ research forward in the United States and elsewhere, we propose that power be more fully and explicitly incorporated in investigating origins and impacts of EQ. Active areas of EQ research so far have focused on measuring EQ as workers’ experiences [18,27,64,65,66,67] and linking poor EQ to poor health at the individual level [16,22,25], but little attention has been given to the specific conditions and mechanisms that create various forms of EQ. Based on the discussion above, we propose that a perspective of power would help clarify the current stagnation in the EQ measurement debate. Below, we offer four approaches for EQ-health research to consider. Table 2 summarizes them and their rationale.

**Approach** **1.** *Using theory to investigate how and why EQ is formed in certain ways and how it contributes to health inequities.*

Theory is critical to the development of research questions and hypotheses, data analysis, and interpretation of findings so that researchers can build a shared understanding [68,69]. Zimmerman [69] observes that most conceptual models in public health describe *what* is involved in a given phenomenon but do not offer explanations as to *why* or *how* it happens. EQ research so far has been focused on *what* EQ is and whether it relates to health; however, to shift the emphasis to *why* and *how* EQ is formed in certain ways, we need theory as our lens to see power. The use of theory helps us to articulate what mechanisms we assume in the relationship between a potential causal factor (e.g., local labor regulations, market concentration) and outcome (e.g., EQ, health inequities) before designing a research project or planning analyses. If the assumed causal mechanism is found to be plausible, then the study can directly point to where and how to intervene.

Tapping into the rich tradition of studying power in philosophy, sociology, psychology, and political science, researchers interested in EQ and health could draw from various theories that are useful in investigating power, EQ, and health; such as theories that reveal how power emerges (status construction theory [53,54]) and how it operates (power resource model [20]), how power shapes life opportunities including health (fundamental causes theory [70]), health power resource theory [71], how power influences individual health-related decision-making (Zimmerman’s multi-level theory [38]), and how power can be challenged and changed (discursive institutionalisms [72]). Relying on theory also facilitates the investigation of other existing power structures such as sexism and racism and their relations to EQ. Institutional racism [73], structural sexism [74], structural intersectionality [35], the constrained choice theory [75], and class relations explaining health inequity [58] are some examples that could be useful in connecting power, marginalization, EQ, and health. Occupational health science training programs may not systematically introduce these theories to students, as these theories are from a wide range of academic disciplines; therefore, interdisciplinary collaboration is crucial in studying EQ. A better understanding of these power structures and EQ could help clarify how EQ may be associated with health inequity and, in turn, suggest a way to reduce health inequity through improving EQ. In addition, clearly articulated, theory-based research focusing on occupational health could contribute to other academic disciplines that may not typically apply their theories to employment and health.

**Approach** **2.** *Investigating the multi-level contexts in which EQ is embedded using a variety of tools and study designs.*

As Lipscomb and colleagues [39] wrote, “understanding the context of work, including how people come to do the work they do and the formal and informal policies under which they toil, is a prerequisite to understanding how work influences health disparities” (p. 41). Because power in social contexts is often invisible in individual-level data commonly used in EQ studies, it takes institutional [3] and multi-level [38] perspectives to incorporate power in EQ research. There are various tools for investigating EQ in social contexts at different levels, ranging from national and local policies to organizational activities and workplace environments. Focusing on national contexts, researchers may take a political economy approach to public health [76] and conduct international comparisons. This line of research typically uses welfare state typologies, or groupings of nations that have similar systems of providing services such as medical care and income replacement in non-working periods [36,59,77]. Previous studies that used welfare state types as contexts for relationships between EQ and health have found unclear results [78,79], but these typologies were not developed specifically to capture how power shapes EQ in each society. EQ researchers may explore different welfare state typologies using indicators such as employment protection, unionization, income replacement, job creation, and employment incentives [80,81,82].

The political economy approach can also be applied to smaller units of analysis. Instead of national policies, researchers may focus on key indicators of power balance that vary across geographical and political units (e.g., states, counties, cities, worksites), industries and occupations, and time periods. Such indicators in the United States may include state and local minimum wages, paid leave policies, right-to-work legislation (i.e., joining union is not a requirement to work in a unionized workplace), state regulations on occupational safety and health, union density, and access to and extent of unemployment benefits. These could capture the specific contexts in which workers experience EQ as well as the contexts in which EQ may be linked to health. Researchers can stay alert to local policy changes as they can offer unique opportunities for investigating what prompted the change, comparing EQ and health before and after, and recognizing unintended consequences on EQ, health, or other social conditions. Public policy scholars conduct this type of research focusing on policy characteristics and health, e.g., [83]. By collaborating with them, EQ researchers could examine if and how these policies influence the power balance that determines EQ.

In addition to policy-level data, gathering information about work organizations and industry practices is a way to facilitate a perspective of power at the organizational level [84]. EQ researchers could explore purposeful monitoring possibilities, as Mialon and colleagues [85] propose, to identify power that large corporations use to reduce labor costs, which shapes EQ [50]. The perspective of power would offer avenues for investigating various forms of self-employment and small businesses, including family farms, in the framework of EQ if researchers address power relations between these diverse forms of work and large corporations.

At the workplace level, individual and multiple case studies as well as intervention studies could investigate the context in which various forms of EQ evolve and how they relate to worker health. These studies may be small in scale, but the richness of data can offer opportunities for clearer understanding of how power imbalances take tangible forms in organizations and local populations. For example, flexible work arrangements in time, and place could be investigated to see if they truly benefit workers [86]. Kelliher and Anderson [87] reported that, even when initiated by workers, flexible work arrangements may intensify the effort exerted during work because case-by-case accommodation for individual workers’ needs generate a sense of indebtedness, which is a form of power imbalance between the worker and the employer. The interconnectedness of EQ dimensions, such as employment stability and pressure to be available for unpredictable hours, which in turn is connected to pay adequacy [33], may become clearer in an intervention study that makes scheduling predictable and carefully documents changes that follow the intervention, both in other EQ dimensions and in health. These research strategies offer in-depth understanding of contexts, subjective experiences, and consequences of EQ.

**Approach** **3.** *Seeking conceptual alignment in EQ measures and refining the concept of EQ within context.*

Although many EQ researchers regret a lack of consensus on the definition of EQ, there is in fact a considerable agreement across conceptual models [4,5], proposed dimensions [10], and efforts to measure EQ [64,65,67]. Researchers agree on *what is relevant to EQ*: political power, welfare state provisions, labor legislation and regulations as antecedents or contexts for EQ, and hazardous working conditions as a consequence. Researchers also generally agree on *what characterizes EQ*: employment stability, workers’ rights in the workplace and the ability to exercise them, and terms of employment (e.g., pay, benefits, hours). Differentiating between what is relevant to EQ and what characterizes it also reminds us to distinguish *work-task intrinsic characteristics* or *work quality*, with which EQ constitutes the broader concept of job quality [11,12]. How best to measure these concepts is likely to vary depending on the context; therefore, aiming for conceptual rather than literal alignment when measuring EQ may be more fruitful.

One example that helps illuminate this suggestion is medical care insurance. If it is obtained through employment, is it workers’ right, or an optional benefit? This depends on the labor law specifying employers’ responsibilities. In contrast, if medical care insurance is not tied to employment and thus not an EQ characteristic, is it still relevant to the relationship between EQ and health? Without larger social contexts, we cannot determine the meaning of medical care insurance in relation to EQ and health. Therefore, seeking a set of context-free questionnaire items that could be used internationally or even nationally may not be most feasible or meaningful, at least until a contextualized understanding of EQ in various societies is developed, which might suggest some common EQ indicators. Relatedly, there remains important opportunity for research that better captures workers’ lived reality of EQ in specific contexts. A powerful tool for “shedding a light on the complex mental processes that are associated with employment experience” [6] (p. 623) is qualitative research because it has inherent strengths in identifying *how* and *why* some aspects of employment are important. Qualitative studies could help not only to clarify what constitutes EQ within distinct contexts, but also continue to refine the conceptual alignment of what characterizes better and worse forms of EQ across societies.

In addition, analyses of existing survey data may contribute to clarifying the EQ concept if they are augmented by theory and contextual data (e.g., state legislature data, historical unemployment data) so as not to decontextualize EQ from power dynamics. In this process, researchers may identify important information that could enhance their analyses such as standardized industry and occupation codes, geographic codes at different levels (e.g., state, county, census tract), other linking systems across various databases, and longitudinal data. Documenting what information would be useful for EQ research would help improve the existing surveillance programs for better quality EQ research in the future.

**Approach** **4** . *Studying EQ as a structural determinant of health inequity in the United States.*

US researchers have recently joined their colleagues in Europe and elsewhere to continue developing the literature on EQ and health. As the conversation on EQ has just begun in the United States, there are many opportunities to explore and situate workers’ experience of EQ within the US sociopolitical and economic reality. US researchers have the advantage of learning from previous research, but a perspective of power highlights the importance of a context-specific understanding of EQ. The approaches we laid out above—using theory, focusing on contexts, seeking conceptual alignments rather than literal agreements—may help build momentum for EQ research in the United States to go beyond the current difficulties in EQ measurements. This next generation of studies could be more compelling if the relationship between EQ and health is described with clear, theory-based causal assumptions linking specific social contexts to EQ and its impacts on health [68,88,89].

One particular area to which EQ researchers in the United States could make unique contribution is health inequalities. By investigating EQ as a structural determinant of health, EQ researchers could clarify the interdependent nature of occupational segregation and EQ from a perspective of power: that is, poor EQ jobs are more likely to be held by marginalized people [24,25,26,27], and to the extent that EQ is associated with health, poor health resulting from poor EQ may explain health inequalities. To test this proposition empirically, EQ studies need to focus on structural forces around employment, such as structural racism [73], structural sexism [74], and structural intersectionality [35], rather than individual workers’ characteristics.

**Table 2:** Approaches to incorporate a perspective of power in the research on employment quality (EQ) and health.

Approach	Rationale
1. Using theory to study how EQ is formed and related to health/health inequity	To organize research endeavors and build shared language and understandings of complex and dynamic phenomena [68,69]. The use of theory is important in exploratory, explanatory, and confirmatory studies [90].
2. Expanding focus from individuals to social contexts in which individuals experience EQ	Relying exclusively on individual-level data obscures the contexts that create power, or lack thereof, which in turn shape health inequity [3,38,51].
3. Measuring EQ conceptually in context	Conceptual agreement exists on what is relevant to EQ and what characterizes it [4,5,10,64,65,67], but variables to best represent these will be specific to time, place, and societal structure. Conceptual, rather than literal, alignment may be more informative.
4. Studying EQ as a structural determinant of health inequity	EQ patterns across individual or worker group characteristics [24,25,26,27] suggest that poor EQ may explain health inequity. Focusing on social structure and processes that distribute EQ unevenly may be useful in understanding health inequity.

## 6. Conclusions

In this paper, we advocate for a perspective of power in order to advance research on EQ, health, and health equity. As Frederick Zimmerman wrote, “to ignore power would be to ignore the most important determinant of population health” [38] (p. 50). Power, however, can be difficult to recognize in research unless we make a conscious effort to do so. The difficulty is partly because public health research has been generally focused on individual-level characteristics and behaviors [91]; accordingly, available data and analytic approaches are more readily useful for answering individual-focused research questions rather than institution- or process-focused ones. The US researchers’ current struggle in operationalizing EQ may be overcome by incorporating a perspective of power in their research, rather than a more common practice of individual-focused, context-free, and power-blind approach to studying work, health, and inequality. Occupational safety and health research has been shifting to expand its focus beyond individual workers’ characteristics and behaviors, as evidenced by the Healthy Work Design and Well-being Program in NISOH (https://www.cdc.gov/niosh/programs/hwd/default.html, accessed on 4 May 2022). This program aims to enhance worker safety and health through structural improvements in work environments and identify the study of precarious employment and non-standard work arrangements as part of the NIOSH strategic plan [30]. The allegory we proposed may facilitate research that investigates these poor forms of EQ from a perspective of power.

Work is a social institution shaped by power [8,39,40,41]; therefore, one important way to understand work’s impacts on health is through a clearer understanding of EQ as a product of power balance. By conducting power-centered research on EQ and health, researchers would be in a position to inform policy interventions to correct power imbalances so that work could be a process of rectifying, rather than exacerbating, health inequity.

## Figures and Tables

**Table 1:** Summary of concepts used in the allegory of employment quality (EQ).

Concept	Definition
Sociopolitical factors	Labor laws and enforcement, workers’ rights and social protections, prevailing business practices, science and technology, and political rhetoric/public discourse [39,40,41,52]. They exist everywhere but their forms and functions are specific to place and time. Together with labor market contexts, they set the grade of the tilted playing field on which the arrangements and conditions of employment exist.
Labor market context	The particulars of time and place-specific unemployment rates, informal employment rates, and demands for goods and services that are interrelated to sociopolitical factors and form the worker–employer reality where arrangements and conditions of employment exist.
Political power	Power used to influence society through the political process, such as employers’ lobbying and political contributions, and workers’ voting and community activism [40]. Political power influences the grade of the slope of the employment playing field.
Power resources [20]	Tools, or sources of power, that the employer and workers each can use to try to achieve their respective wants. Workers can use their human capital (i.e., education, skills) and collective organization. Employers can use hiring and firing authority, job simplification, flexible staffing practices, non-union positions, relocation, and outsourcing. These are sources of potential power and may not be actually exercised (see “power resources model”).
Employment quality	The result of the power dynamics that shapes arrangements and conditions of employment; a package consisting of employment stability, rights for workers and their ability to exercise them, and the terms of employment [4,5,10]. The package exists on a conceptual spectrum of better and poorer configurations of these characteristics. From the employer’s perspective, the package represents labor cost and worker productivity [50].
Power resources model [20]	Describes the calculations and processes parties use in attempting to achieve their respective wants; results in exchange or conflict if power resources are relatively balanced, and exploitation if one party’s power resources outweigh the other’s. Power may not have to be actively used in a given circumstance if all parties understand the imbalance [20,38].

## Data Availability

Not applicable.
